# Symptom practice guide for telephone assessment of patients with cancer treatment-related cardiotoxic dyspnea: Adaptation and evaluation of acceptability

**DOI:** 10.1186/s40959-017-0026-6

**Published:** 2017-12-28

**Authors:** F. Kelly, S. L. Carroll, M. Carley, S. Dent, R. Shorr, J. Hu, R. Morash, D. Stacey

**Affiliations:** 1 School of Nursing, University of Ottawa, 451 Smyth Road, Ottawa, Ontario K1H M5 Canada; 2 Clinical Epidemiology Program, Ottawa Hospital Research Institute, 501 Smyth Road, Room 1280, Box 201B, Ottawa, Ontario K1H 8L6 Canada; 3 School of Nursing, McMaster University, 1280 Main Street West, Room HSC2J40, Hamilton, Ontario L8S 4K1 Canada; 4 The Department of Medicine, University of Ottawa, 451 Smyth Road, Ottawa, Ontario K1H M5 Canada; 5 The Ottawa Hospital General Campus, 501 Smyth Road, Ottawa, Ontario K1H 8L6 Canada

**Keywords:** Symptom practice guide, Cancer treatment-related cardiotoxicity, Self-management/care

## Abstract

**Background:**

Patients with cancer treatment-related cardiotoxicity, which may manifest as heart failure (HF), can present with dyspnea. Nurses frequently assess, triage and offer self-care strategies to patients experiencing dyspnea in both the cardiology and oncology settings. However, there are no known tools available for nurses to manage patients in the setting of cancer treatment-related cardiotoxicity. The objective of this study was to adapt and evaluate the acceptability of an evidence-informed symptom practice guide (SPG) for use by nurses over the telephone for the assessment, triage, and management of patients experiencing dyspnea due to cancer treatment-related cardiotoxicity.

**Methods:**

The CAN-IMPLEMENT© methodology guided this descriptive study. A systematic search was conducted in four databases to identify cardio-oncology and HF guidelines and systematic reviews. Screening was conducted by two reviewers, with data extracted into a recommendation matrix from eligible guidelines and systematic reviews on: assessment criteria, medications, and/or self-care strategies to manage dyspnea. Healthcare professionals with an expertise in oncology and/or cardiology were recruited using purposeful and snowball sampling. Evaluation of acceptability of the adapted SPG was gathered through semi-structured interviews and a survey with open- and closed-ended questions. Quantitative findings and participant feedback from the interviews and the open-ended survey questions were analyzed descriptively.

**Results:**

Of 490 citations, seven HF guidelines were identified. Evidence from these guidelines was added to the original SPG. Eleven healthcare professionals completed the interview and acceptability survey. The adapted SPG was iteratively revised three times during the interviews. The original SPG was adaptable, and participants indicated the adapted SPG was comprehensive, easy to follow, and would be useful in clinical practice.

**Conclusions:**

This study highlights the lack of knowledge tools and available clinical practice guidelines to guide healthcare professionals to assess, triage and/or offer self-care strategies to patients with cancer treatment-related cardiotoxic dyspnea. Moreover, most nurses require assistance to differentiate among the various causes of dyspnea from oncology treatment in order to triage severity appropriately. Further research should focus on evaluating the validity of the adapted SPG in clinical practice.

**Electronic supplementary material:**

The online version of this article (10.1186/s40959-017-0026-6) contains supplementary material, which is available to authorized users.

## Background

With the delivery of more complex cancer interventions, including chemotherapy, targeted therapies and radiation treatment, cancer treatment-related cardiotoxicity has emerged as a potential consequence [1–3]. Cardiotoxicity is defined as the direct effects of cancer treatment on heart function and structure, and is the second leading cause of long-term morbidity and mortality among those treated with cancer therapies [4, 5]. Dyspnea is one symptom patients with cancer treatment-related cardiotoxicity may experience [2].

Dyspnea is defined as a subjective feeling of breathing discomfort or difficulty that may vary in intensity, and is a symptom patients treated with cancer therapies may experience [6, 7]. Dyspnea is associated with lower levels of physical performance and decreased social functioning, thereby negatively affecting the overall quality of life of adults treated with cancer therapies [8–10]. Dyspnea may also indicate a more severe complication of cancer and its treatment, such as heart failure (HF), pulmonary embolism, and anemia.

Nurse-led support is offered in both the cardiology setting, with regards to patient self-management of HF, and the oncology setting. Nurses can assess and triage patient’s experiencing dyspnea before it becomes potentially life threatening, and they may assist in the development of strategies to help alleviate the distress patients experience with dyspnea [11, 12]. Assessment, triage and guidance in self-management of dyspnea in such patients can occur over the telephone from ambulatory clinics [13].

Clinical outcomes for HF depend largely on patient self-management [14]. Inadequate symptom monitoring and treatment during exacerbations may result in patients being hospitalized [14]. To avoid this, in some settings, remote patient monitoring systems and cost-effective disease management strategies have been established [14]. A previous randomized controlled trial evaluated the use of nurse-led telephone monitoring (intervention) versus usual care [15]. Nurses used predetermined standardized questions to assess a variety of signs and symptoms, such as dyspnea, daily weight monitoring, drug adherence, and physical activity [15]. Based on their assessment, nurses were then able to either adjust medical therapy, such as dose of diuretic in patients with HF, or recommend a nonscheduled medical visit [15]. Those in the intervention group had fewer re-hospitalizations both in the short term, and one to three years following the intervention [15, 16].

To support oncology nurses with the assessment, triage, documentation and guidance of patients to self-manage their cancer treatment-related symptoms, the pan-Canadian Oncology Symptom Triage and Remote Support (COSTaRS) team developed and evaluated 15 evidence-based symptom practice guides (SPGs) [17]. The COSTaRS SPGs have been implemented in multiple ambulatory oncology programs across Canada, and their uptake has been previously evaluated in three different healthcare systems [18]. One of the SPGs produced by the COSTaRS team was for dyspnea [17]. The original COSTaRS Dyspnea SPG consisted of five recommendations for the nurse to (a) assess symptom severity, (b) triage a patient for symptom management based on the highest assessment item of severity, (c) review medications being used for the symptom, (d) review self-management strategies, and (e) summarize and document the plan agreed upon with the patient [19]. The original COSTaRS Dyspnea SPG, however, did not include clinical practice guidelines or systematic reviews with a focus on symptom management for dyspnea due to cancer treatment-related cardiotoxicity.

The original COSTaRS Dyspnea SPG was chosen to be reviewed and adapted for patients experiencing cancer treatment-related cardiotoxicity given that new and spontaneous reporting of dyspnea is one symptom that may present in these patients [20]. Dyspnea in those experiencing cancer treatment-related cardiotoxicity may be non-specific and should therefore be triaged appropriately [20].

The objective of this study was to adapt and evaluate the acceptability of an evidence-informed SPG for use by nurses over the telephone for the assessment, triage, and management of patients experiencing dyspnea due to cancer treatment-related cardiotoxicity. The research questions were: (1) what adaptations were required when cardiology clinical practice guidelines were added to the original COSTaRS Dyspnea SPG?; and (2) was the adapted dyspnea SPG acceptable for oncology nurses to use when providing symptom support?

## Methods

The CAN-IMPLEMENT© methodology guided this descriptive study. CAN-IMPLEMENT© recommends six steps for knowledge tool adaptation: (1) a call to action; (2) plan; (3) search and screen; (4) assess and select; (5) draft, revise and endorse recommendations; and (6) obtain user-centered feedback to plan implementation [21]. Ethics approval for this study was received from the Ottawa Health Science Network Research Ethics Board (20160752-01H) and the University of Ottawa Research Ethics Board (A12–16-02). All participants provided written informed consent.

### Step one: A call to action

Step one involves clarifying the incentive for the adaptation [21]. The incentive for this study was the increasing prevalence of patients with cancer treatment-related cardiotoxicity and the lack of tools available for nurses to use to assist this patient population with symptom self-management.

### Step two: Plan

Step two includes establishing the scope of the knowledge tool, determining the feasibility of the adaptation, forming an organizing committee, and writing the work plan [21]. For this study, the research question was: what adaptations were required when cardiology clinical practice guidelines were added to the original COSTaRS Dyspnea SPG? A research team was formed consisting of experts in cardio-oncology (SD), cancer survivorship (RM), and SPG development (DS). A research proposal was then established.

### Step three: Systematic search and screen

Step three is searching and identifying eligible guidelines related to the specified topic [21]. We conducted a systematic search of the available literature for clinical practice guidelines and systematic reviews about assessing, triaging and offering self-management strategies for adults experiencing dyspnea due to cardiotoxicity or HF. The search strategy was designed in collaboration with a health science librarian (RS) and was based on the strategy used for the COSTaRS SPGs [19]. The systematic search was reported to meet the Preferred Reporting Items for Systematic Reviews and Meta-Analyses (PRISMA) criteria [22]. The search focused on all key databases relevant to the subject matter: Medline, Cochrane Database of Systematic Reviews, CINAHL and Web of Science. Articles were searched from January 2010 until December 2016 to identify current evidence. To supplement the database search, grey literature searches were also conducted on websites known or suspected to have cardiology practice guidelines related to symptom self-care and known guideline clearinghouse websites (Appendix A) [19, 23].

Inclusion/exclusion criteria were established using the PIPOH framework (Population, Intervention, Professionals/Patients, Outcomes and Health Care Setting) (see Table 1) [19, 24]. Eligible citations were systematic reviews and clinical practice guidelines evaluating cardiac-related symptom interventions to assess, rate severity and/or manage dyspnea in adults with cardiotoxicity and/or HF.

**Table 1:** The eligibility criteria for the symptom practice guide – dyspnea

	Inclusion	Exclusion
Population	Adults, defined as 18 years of age or older, with cardiotoxicity and/or heart failure.	Children aged 17 years of age or younger.
Intervention	Cardiac related symptom intervention to assess, rate severity, or manage dyspnea.	–
Professionals targeted	Nurses and other healthcare professionals working in oncology and/or cardiology services.	–
Outcomes	Appropriate referrals for medical consultation, safe management of symptoms, and patients guided in self-care.	–
Healthcare setting	Telephone patients at home receiving services through an ambulatory oncology program.	–
Methodology	Clinical practice guideline Systematic review	Randomized control trial Cohort study Pre−/post-test study Case-control study Cross-sectional study Case reports and series Editorials, Opinions
Language	Any	–
Publication Dates	2010 or later	Prior to 2010

### Step four: Assess and select

Step four includes assessing the quality, content, consistency, acceptability and applicability of the guideline recommendations [21]. After duplicates were removed, two reviewers independently conducted three levels of screening (FK, DS). Level one screening included a title screen to determine citation relevance to the focus of the SPG. Level two screening used the PIPOH criteria to review abstracts. For both level one and level two screening, citations indicated as *excluded* by both reviewers were removed and citations rated as *included, unsure* or *excluded* by one reviewer were included for the next level of screening. Level three used full text screening. For discrepancies at full-text screening, the reviewers met to discuss and reach consensus.

Two reviewers independently appraised the guidelines using the domain of rigour in the Appraisal of Guidelines for Research and Evaluation (AGREE) II Instrument (FK, JH) [25]. The AGREE II Instrument has been proven reliable and valid for assessing the quality of clinical practice guidelines [25, 26]. The quality rating for the included guidelines was calculated using the AGREE II formula. Although the methodological quality of systematic reviews was planned a priori to be assessed using AMSTAR, no systematic reviews were eligible [27].

After selection and appraisal of the identified guidelines, one author independently extracted data (FK), which was then independently audited by a second reviewer (MC). Characteristics that were extracted included the author and the publication year, criteria to assess and triage dyspnea, medications, and self-care items for symptom management. A recommendation matrix was populated with the extracted characteristics to allow for comparison (Additional file 1).

### Step five: Draft, revise and endorse recommendations

Step five of the CAN-IMPLEMENT© methodology involves preparing an adapted knowledge tool for external review [21]. The original COSTaRS Dyspnea SPG was adapted to incorporate evidence from the eligible cardiology guidelines. Data was extracted from the cardiac guidelines using a recommendation matrix. Two co-authors then further reviewed the adapted SPG (DS, SC).

### Step six: Obtain user-Centered feedback to plan implementation

Feedback on the adapted SPG was gathered during interviews with eligible healthcare professionals at The Ottawa Hospital. The hospital is a large academic teaching hospital in Canada, serving a population of 1.3 million people [28]. Purposeful sampling was used, followed by snowball sampling [29]. Those who were purposively sampled included oncology nurses/advanced practice nurses (APNs) (RN-onc) who were familiar with COSTaRS at The Ottawa Hospital Cancer Centre, cardiology nurses/APNs (RN-cardiac), nurse practitioners (NPs) with a focus on cardio-oncology, and physicians (MDs) with an expertise in cardio-oncology from the Ottawa Cardiac Oncology Program.

Participants were interviewed using a semi-structured interview guide, as well as completing an acceptability survey. The interviewer guided the healthcare professional through the adapted SPG and invited the participant to provide feedback. During the interview, healthcare professionals were asked for their first impressions of the adapted SPG, their thoughts regarding its helpfulness for handling symptom calls from cardio-oncology patients, changes that should be made to ensure the adapted SPG was more useful, and whether any cardiology guidelines were missed.

The acceptability survey was completed during the interview. The survey items, taken from previous surveys, were used for implementing COSTaRS in clinical practice and have demonstrated face validity [30, 31]. Participants were asked to rate the amount of information on the adapted SPG, their comfort using the adapted SPG, referring the adapted SPG to others, and the comprehensiveness of the adapted SPG. Demographic information was also collected.

A researcher (FK) digitally recorded the interviews and took verbatim transcription of the recordings. This approach offers a highly rigorous data set with a low risk of error [32]. Iterative changes to the adapted SPG were made based on the feedback from the interviews and the acceptability survey.

### Analysis

Quantitative findings from the acceptability survey were entered into a Microsoft Excel© spreadsheet and analyzed descriptively. Participant feedback from the interviews and the open-ended survey questions were also analyzed descriptively.

## Results

### Systematic search to identify cardiology evidence

**Fig. 1:**
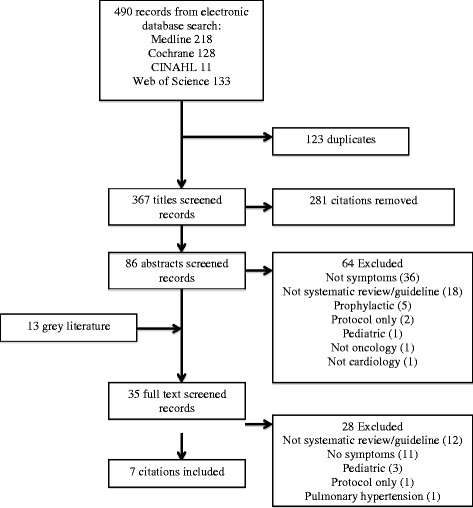
The PRISMA flow diagram. Details of the search and selection process

A total of 490 citations were identified for cardiology-related dyspnea (see Fig. 1). Grey literature searches, including the guideline clearinghouse and websites from cardiac or cardiac-related organizations, identified an additional 13 guidelines. Of the 35 full-text articles screened, seven guidelines for HF were included (see Table 2). The 28 excluded articles were not systematic reviews or guidelines (*n* = 12), had no dyspnea-related assessment, triage or self-care items (*n* = 11), not for adults (*n* = 3), a protocol (*n* = 1), and for pulmonary hypertension (*n* = 1) (see Additional file 2). The eligible HF guidelines were found in peer-reviewed publications (*n* = 4), and on websites of national cardiology organizations (*n* = 3). The AGREE II domain of rigour of development had a median score of 71% (range 23% to 92%).

**Table 2:** Characteristics of included guidelines (*n* = 7)

Author	Country	Year	Title	Quality Rating* (%)
SIGN	Scotland	2016	Management of Chronic Heart Failure	92
American College of Cardiology, the American Heart Association and the Heart Failure Society of America	USA	2016	2016 ACC/AHA/HFSA Focused Update on New Pharmacological Therapy for Heart Failure: An Update of the 2013 ACCF/AHA Guideline for the Management of Heart Failure.	83
American College of Cardiology Foundation and the American Heart Association	USA	2013	2013 ACCF/AHA Guideline for the Management of Heart Failure	81
Canadian Cardiovascular Society	Canada	2012	The 2012 Canadian Cardiovascular Society Heart Failure Management Guidelines Update: Focus on Acute and Chronic Heart Failure	71
National Heart Foundation of Australia	Australia	2011	Guidelines for the Prevention, Detection and Management of Chronic Heart Failure in Australia	62
European Society of Cardiology	Poland	2016	2016 ESC Guidelines for the Diagnosis and Treatment of Acute and Chronic Heart Failure	54
British Columbia Guidelines	Canada	2015	Chronic Heart Failure – Diagnosis and Management	23

*Quality rating for AGREE II domain of rigour development

### Adapting the Dyspnea symptom practice guide

Evidence from seven clinical guidelines was added to the original COSTaRS Dyspnea SPG. Four reviewers (FK, DS, SC, MC) further modified the adapted SPG prior to participant evaluation. These initial changes included: removing assessment items recommended for symptoms of HF not specific to dyspnea (e.g. vomiting/diarrhea, feelings of dizziness or lightheaded, feelings of confusion, and feelings of syncope); removing medications recommended for symptoms of HF not seen as specific for managing dyspnea (e.g. angiotensin-converting enzyme inhibitor, beta-blockers, digoxin, etc.); and removing the self-care recommendation to have an annual influenza vaccine. To enhance ease of readability by healthcare professionals, colour-coding sections specific to *oncology*, *cardiology* and *both* was added, as well as groupings of the items together based on their originating guidelines (oncology, cardiology or both). The assessment items regarding chest pain remained separate as the item recommended by oncology guidelines focused on assessing for a pulmonary embolism, whereas the item recommended by HF guidelines focused on assessing for an acute coronary syndrome event.

### Characteristics of participants

Eleven healthcare professionals were interviewed including oncology nurses/APNs (*n* = 4), cardiology nurses/APNs (n = 4), and cardiologists with an expertise in cardio-oncology (*n* = 3) (see Table 3)**.** The mean age of the participants was 47 years of age and 10 were female.

**Table 3:** Demographic characteristics of participants (*n* = 11)

Characteristics	Oncology Nurses (*n* = 4)	Cardiology Nurses (*n* = 4)	Cardiologists (*n* = 3)
Age (in years)			
30–39 40–49 50–59 60–69	0 1 1 0	2 1 1 0	0 2 0 1
Sex			
Female Male	4 0	4 0	2 1
Highest level of education:			
Undergraduate degree Graduate degree Medicinae Doctor	1 3 0	0 4 0	0 0 3
Role			
Physician Registered nurse Advanced practice nurse Nurse educator	0 1 1 2	0 1 2 1	3 0 0 0
Hours worked			
Full time Regular part-time Casual	3 1 0	3 0 1	2 1 0
Length in current position			
1–2 years 3–5 years 6–10 years ≥ 10 years	1 0 2 1	2 0 1 1	0 0 0 3

### SPG revisions

Three iterative revisions were made to the adapted SPG during the interviews based on participant feedback (see Table 4). The first revision included four changes. For example, the language used to assess patients for tachycardia was unclear. This was supported by the quote “*And then, I’m not sure how I would answer as a patient ‘Do you have a fast heart beat that won’t slow down?’”* (RN-onc.). The adapted SPG was therefore revised to replicate the language used in the BC Guidelines (2015) [33], “Do you have a fast heartbeat that does not slow down when you rest?”

**Table 4:** Revisions to adapted SPG

Participant Comment	Supporting Quotations	Revision
Revision #1		
Chest pain question appears as two separate questions, rather than linked together.	*“So it’s not an automatic question if somebody says no I don’t have chest pain, then you’re not gonna ask them that question”* RN-onc	Revised to “If you have chest pain, does it go away with rest or medication?”
Language used to assess for patients tachycardia is unclear.	*“And then, I’m not sure how I would answer as a patient ‘Do you have a fast heart beat that won’t slow down?’”* RN-onc	Revised to “Do you have a fast heartbeat that does not slow down when you rest?”
Self-care strategy for limiting sodium and fluid intake has unfamiliar units (self-care strategies number 11 and 12).	*“I would have no idea as a layperson if I, when I see less than 2000 mg a day.”* RN-cardiac “*Have you tried limiting your salt intake to, this is a funny number, right? What is 0.4 of a teaspoon of salt?*” MD	Revised to “Have you tried limiting your salt intake to under half a teaspoon (under 2000 mg) per day?” and “Are you aiming for a fluid intake of 6 to 8 cups (1.5 to 2 L) per day?”
Lacking an assessment of smoking and drinking prior to offering self-care strategy (self care strategy numbers 13 and 14).	“*‘Have you tried to stop, uh, to stop smoking or drinking?’ Well how do you know that I do?”* RN-onc	Revised to “If you smoke, have you tried to stop?” and “If you drink more than 1–2 standard alcoholic drinks per day, have you tried to reduce your alcohol intake to 1 drink per day?”
Revision #2		
Lacking something in the title to differentiate adapted SPG from original COSTaRS SPG.	*“even just reflecting in the title of the practice guide that this one would be related to cardiotoxicity.”* RN-cardiac	Revised to “Breathlessness/Dyspnea Practice Guide: Cardiotoxicity”
Missing a way for the nurse to document whether chest pain has gone away with rest and/or medication, and which medication relieved the pain.	*“so should the clinician be able to, to uh, document whether the pain does subside with medication or with rest?”* RN-cardiac	Revised to “If you have chest pain, does it go away with: □ Rest or □ Medication?______________”
Missing a way for the nurse to document how many pillows the patient has increased for sleeping.	*“is it important to note the number of pillows that they have increased?”* RN-cardiac	Revised to “Have you increased the number of pillows you need to sleep? Increase in number of pillows:______”
Missing space for the nurse to document the patient’s description of their dyspnea.	*“So is this space intended for the description?”* RN-cardiac	Revised to “Does your shortness of breath interfere with your daily activities at home and/or at work? Describe:”
Unclear what the coloured boxes are.	*“You might even have a little title that says legend”* RN-cardiac	Revised to “**Legend:** ♥ Cardiology ★ Cardiology and Oncology”
Self-care strategy suggesting exercise requires emphasis on symptom stability.	*“And so, perhaps to start the question, uh, state, when breathlessness is stable.”* RN-cardiac	Revised to “When breathlessness is stable, have you tried 30 min of exercise at least twice a week?”
Lacking space for the nurse to document.	*“I often think of as, the whiteness being paper real-estate”* RN-cardiac	Revised to make margins smaller around document.
Unsure if patient would remember how many pillows they have increased from their baseline.	*“if there was a baseline I like the idea of that”* RN-cardiac	Revised to “Baseline #:______” and “Current #:______”
Lacking space in the self-care strategy for weight management to document the patients’ weight at the time of the call (self-care strategy number 16).	*“the last time you called your weight was”* RN-cardiac	Revised to “Are you weighing yourself daily (after waking and voiding, before dressing and eating)? Weight______”
Revision #3		
Missing assessing for paroxysmal nocturnal dyspnea.	*“I notice you don’t ask about, um, PND though in here, right?”* MD	Revised to “Are you waking up at night with shortness of breath?”
Lacking space for nurse to document if patient is unsure whether he/she have gained or lost weight at time of the call.	*“I find that unless they know about heart failure and you’ve had, they’ve already had teaching on it they’re probably not weighing themselves everyday”* MD	Revised to “Have you gained or lost weight in the last week? □ Unsure”
Lacking nitrates to assist with dyspnea.	*“nitrates also help heart failure so”* MD	Revised to “Nitrates – Benefits Balanced With Harm”
Printer only prints in black-and-white rather then in colour.	*“and then I saw the colours because this I thought, my first comment was ‘What’s this?’”* RN-onc	Revised to ♥ for cardiology evidence, and ★ for both cardiology and oncology evidence

*RN-onc* oncology registered nurse/advanced practice nurse, *RN-cardiac* cardiology registered nurse/advanced practice nurse, *MD* physician

The second revision involved nine changes. For example, adding tick-boxes to indicate whether chest pain went away with either rest or medication, and adding space to write which medication relieved the pain, if applicable. This was supported by the quote “*So should the clinician be able to, document whether the pain does subside with medication or with rest?”* (RN-cardiac.) The adapted SPG was therefore revised to “If you have chest pain, does it go away with: □ Rest or □ Medication?____________”.

**Fig. 2:**
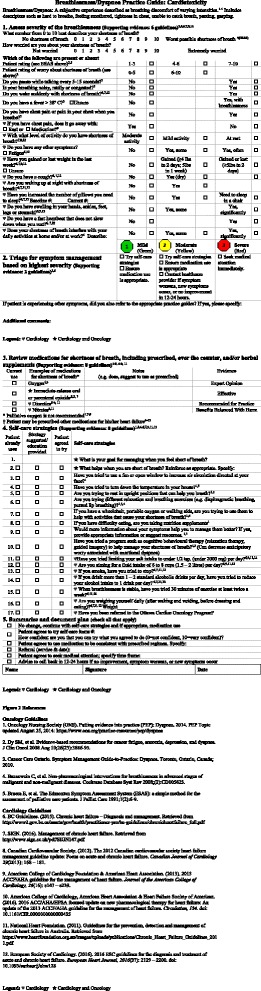
Breathlessness/Dyspnea Practice Guide: Cardiotoxicity. The adapted version with evidence added from seven heart failure guidelines

The third revision had four changes. For example, adding the assessment question regarding paroxysmal nocturnal dyspnea. This was supported by the quote “*I notice you don’t ask about PND though in here, right?”* (MD.) The adapted SPG was therefore revised to “Are you waking up at night with shortness of breath?” (see Fig. 2).

### Adapted SPG acceptability

Participants evaluated the acceptability of the adapted SPG during the interviews (see Tables 5 and 6). Most participants (*n* = 10) found the SPG understandable and all participants stated they would be comfortable or very comfortable telling someone about the adapted SPG as a resource to assist adults with cardiotoxicity with the self-management of their dyspnea. Further suggestions included: (1) use the adapted SPG with cancer survivors in the primary healthcare setting; (2) develop a pocket version of the adapted SPG; and (3) make the adapted SPG into a mobile application.

**Table 5:** Participant acceptability of the adapted SPG based on profession (*N* = 11)

		RN-onc *N* = 4	RN-cardiac *N* = 4	MD *N* = 3
How comfortable would you be using the SPG?	Not very comfortable Uncomfortable Neutral Comfortable Very comfortable	- - - 4 -	- - - 4 -	- - - 3 -
How comfortable would you be telling someone?	Not very comfortable Uncomfortable Neutral Comfortable Very comfortable	- - - 2 2	- - - 2 2	- - - 3 -
Information on the SPG	Too much information Just right Not enough information	- 3 1	- 4 -	- 3 -
Is the SPG understandable?	Yes No	4 -	3 1	3 -
Were any guidelines missed?	Yes No	- 4	- 4	- 3

*RN-onc* oncology registered nurse/advanced practice nurse, *RN-cardiac* cardiology registered nurse/advanced practice nurse, *MD* physician

**Table 6:** Participant acceptability of the adapted SPG based by revision (*N* = 11)

		Revision 1 *N* = 4	Revision 2 *N* = 3	Revision 3 *N* = 4
How comfortable would you be using the SPG?	Not very comfortable Uncomfortable Neutral Comfortable Very comfortable	- - - 4 -	- - - 3 -	- - - 4 -
How comfortable would you be telling someone?	Not very comfortable Uncomfortable Neutral Comfortable Very comfortable	- - - 3 1	- - - 2 1	- - - 2 2
Information on the SPG	Too much information Just right Not enough information	- 3 1	- 3 -	- 4 -
Is the SPG understandable?	Yes No	4 -	2 1	4 -
Were any guidelines missed?	Yes No	- 4	- 3	- 4

First impressions of the healthcare professionals from the interviews were that the adapted SPG was comprehensive and easy to follow:

*Very positive. I thought it was excellent. This cardio-oncology is all new to me. As I said I’ve never worked in it so I was really impressed there was a guide like this in place. I thought this was far more comprehensive, as I said, than the other one* [original COSTaRS Dyspnea SPG] *and easier to walk through with far more detail.* (RN-cardiac.)

Participants also liked having the evidence colour-coded and grouped together based on origin of the evidence:

*…colour-coding, that was a smart thing to do. So then if it’s anything like this you’re going towards interventions for shortness of breath. And then if it’s anything in there then you’re going for interventions around cardiac toxicity* (RN-onc.)

Participants further believed the adapted SPG would be helpful for handling symptom calls from patients with cardiotoxicity:

*I think so. I absolutely do because I think whoever’s on the other end, which will be an oncology nurse, but we have to realize that many of these patients are on potentially cardiotoxic treatments.* (MD.)

## Discussion

The CAN-IMPLEMENT© methodology used for developing the original COSTaRS Dyspnea SPG was replicated when adapting the SPG to include cardiology guidelines. A systematic and rigorous approach was used to search and screen for available guidelines, to extract data using a recommendation matrix, and to be transparent in how the evidence was used to inform the adapted SPG. Participant feedback led to three iterative revisions of the adapted SPG. Participants found the adapted SPG understandable, comprehensive, easy to follow, and believed it would be helpful for handling symptom calls from patients with dyspnea due to cancer treatment-related cardiotoxicity. These findings lead to three areas of discussion.

First is the apparent lack of clinical guidelines or systematic reviews with available tools for nurses to assess, triage and/or offer self-care strategies to their patients with cardiotoxic-related symptoms. Cardio-oncology is a relatively new field of study. In the last year a small number of guidelines were published, however, these guidelines tended to focus on the diagnosis and medical management of cardiotoxicity, rather than offer self-care management recommendations for nurses to support their patients with symptom management [5, 34–36]. As a result, the scope for the systematic search for cardiology evidence was expanded to include HF guidelines. Nurses are appropriate healthcare professionals who possess the requisite knowledge and skill to inform, motivate and assist patients in the successful self-management of their chronic illness, such as dyspnea related to long-term cardiotoxicity [37]. However, nurses require user-friendly evidence-informed tools to help them guide patients in self-management [37].

Our study highlights the need for healthcare professionals, across areas of specialties, to collaborate and share their expertise. In the Ottawa Cardiac Oncology Program, physicians with expertise in cardiology and oncology collaborate to provide comprehensive cancer care while trying to avoid long-term cardiotoxicity related to cancer treatment [38]. However, the clinic does not have nurses available to respond to patient concerns regarding cardiotoxicity. Therefore, the nurses at The Ottawa Hospital who typically respond to telephone calls from patients experiencing cardiotoxic-related dyspnea are oncology nurses with limited cardiology knowledge. Nurses providing care to patients with cardiotoxic symptoms require additional, evidence-based cardiology knowledge to guide patients in their symptom management. Knowledge tools, like SPGs, can bridge this gap. Although the original COSTaRS Dyspnea SPG offered assessment, medication and self-care items based on oncology evidence, it neglected to include cardiology evidence, thereby overlooking recommendations for dyspnea management in the setting of cancer treatment-related cardiotoxicity. As cardiotoxicity commonly presents itself as HF, the need to safely triage symptoms, such as dyspnea, to the appropriate level of care is of particular importance. HF, regardless of its origin, is the leading cause of hospitalization for those 65 years of age and over, with each hospital admission costing between $6000 and $15,000 [39]. With the additional self-care strategies in the adapted SPG, oncology nurses may safely assist their patients in managing their dyspnea in their home until they are able to appropriately see their healthcare provider. As oncology nurses may be the first line of contact for cancer patients, it is important that the self-care items they are offering patients are evidence-based, as represented by the adapted SPG.

Moreover, nurses require assistance to differentiate among the various causes of dyspnea in order to triage severity appropriately [40]. As a first point of contact, participants in our study indicated the importance of using the assessment questions to assist oncology nurses in differentiating among the various causes of dyspnea, thereby allowing them to appropriately triage their patients. It has been previously described that collaboration between nurses and physicians is strengthened when nurses’ concerns are based on case knowledge, the scientifically established knowledge that allows physicians to make medical diagnoses [40]. The colour-coding sections specific to *oncology*, *cardiology* and *both*, changed to symbols in the third revision, was highly rated by participants as it allowed clearer guidance with regards to which questions indicated a cardiac cause of dyspnea versus other potential causes of dyspnea (i.e., infection, anemia, pulmonary embolism, etc.). As patients treated for cancer may experience a number of symptoms from a multitude of causes, nurses require support in differentiating among such causes in order to safely triage patients over the phone.

### Strengths and limitations

To improve quality and reduce the risk of bias: (a) the protocol for the systematic search was developed a priori; (b) a comprehensive search of four electronic databases and grey literature was conducted; and (c) two reviewers screened the identified literature and appraised the quality of the included studies [27, 41, 42].

Semi-structured interviews were used to introduce participants to the adapted SPG and offered participants the freedom to respond to questions in their own words with as much detail as they desired [29, 32]. We were able to interview various healthcare professionals, however were unable to recruit an oncology nurse who specialized in cardio-oncology, given the novelty of this sub-specialty. Moreover, the adapted SPG was developed and tested in one Canadian site. Further evaluation is required to ensure the adapted SPG is applicable for use in other ambulatory oncology programs. Nevertheless, confidence may be warranted that the adapted SPG is generalizable for use at other sites given the uptake of the original COSTaRS Dyspnea SPG has previously been studied in three different healthcare settings.

## Conclusion

Guided by the CAN-IMPLEMENT© methodology, it was possible to adapt the original COSTaRS Dyspnea SPG by adding evidence from seven HF guidelines. User-centered feedback led to the adapted SPG being iteratively revised three times during the interviews. Healthcare professionals found the adapted SPG comprehensive and easy to follow, and believed it would be useful in clinical practice.

Future research should focus on evaluating the validity of the adapted SPG for identifying dyspnea due to cancer treatment-related cardiotoxicity in clinical practice. Validation of the adapted SPG would focus on its predictive validity, a type of construct validity [43]. Evaluating the predictive validity of a triage tool is the most frequently used method for assessing their validity, and it considers the degree to which the triage acuity level is able to predict the true acuity of the patient [43, 44].

### Additional file

Additional file 1: The recommendation matrix of the included guidelines (n=7). (DOC 91 kb)

Additional file 2: Excluded studies (n=28). All citations listed below were reviewed in their full-text version and excluded for the reason indicated. An alphabetical reference list follows the table. (DOC 89 kb)

## Appendix

**Table 7:** The search strategy used for Medline

1	Exp Neoplasms/ and Survivors/
2	(cancer adj10 survivor*).tw.
3	1 or 2
4	Cardiotoxicity
5	Exp cardiomyopathies/ci [Chemically Induced]
6	(cardiotoxic* or card* toxic*).tw.
7	4 or 5 or 6
8	3 and 7
9	Guideline/ or practice guideline/
10	Guidelines as topic/ or practice guidelines as topic/
11	(guideline or practice guideline or consensus development conference or consensus development conference, NIH).pt.
12	(position statement* or policy statement* or practice parameter* or best practice*).tw.
13	(standards or guideline or guidelines).ti.
14	((practice or treatment* or clinical) adj guideline*).ab.
15	(CPG or CPGs).ti.
16	Consensus*.ti.
17	((critical or clinical or practice) adj2 (path or paths or pathway or pathways or protocol*)).tw.
18	(care adj2 (standard or path or paths or pathway or pathways or map or maps or plan or plans)).tw.
19	Clinical protocols/
20	Critical pathways/
21	Consensus/
22	(MEDLINE or systematic review).tw. or meta analysis.pt.
23	Or/9–22
24	8 and 23
25	7 and 22

## Abbreviations

AGREE: Appraisal of Guidelines for Research and Evaluation
APN: Advanced practice nurse
COSTaRS: pan-Canadian Oncology Symptom Triage and Remote Support
HF: Heart failure
MD: Physician
NP: Nurse practitioner
PIPOH: Population, Intervention, Professionals/Patients, Outcomes and Health Care Setting
PRISMA: Preferred Reporting Items for Systematic Reviews and Meta-Analyses
RNAO: Registered Nurses’ Association of Ontario
RN-cardiac: cardiac registered nurses/advanced practice nurse
RN-onc: oncology registered nurse/advanced practice nurse
SPG: Symptom practice guide
