# Supplementary material for: Symptom practice guide for telephone assessment of patients with cancer treatment-related cardiotoxic dyspnea: Adaptation and evaluation of acceptability
Source: Cardiooncology. 2017 Dec 28;3:7. doi: 10.1186/s40959-017-0026-6 (PMC7048126; doi:10.1186/s40959-017-0026-6)
Supplement: Supplementary file 2 — Excluded studies (n=28). All citations listed below were reviewed in their full-text version and excluded for the reason indicated. An alphabetical reference list follows the table. (DOC 89 kb) [file 40959_2017_26_MOESM2_ESM.doc]

Appendix B

The recommendation matrix of the included guidelines (n=7).

| **Author** | **Year** | **Assess** | **Triage** | **Medications** | **Management** |
| --- | --- | --- | --- | --- | --- |
| 6. BC Guidelines | 2015 | Weight gain (2kg over 2 days or 2.5kg in one week) (p. 2)  No chest discomfort, pressure or pain (p. 4)  You have a dry hacking cough (p. 4)  You have chest pain that does not go away with rest or medication (p. 4)  See “Heart Failure Zones” (p. 4) | See “Heart Failure Zones” (p. 4) | Furosemide (p. 5, 6)  Angiotensin converting enzyme inhibitor (ACE-I) (or angiotensin receptor blocker [ARB], if ACE-I intolerant) (p. 5)  Beta-blocker (BB) (p. 6)  Mineralocorticoid receptor antagonists (MRA) (p. 6)  Digoxin is no longer considered a first-line therapy for heart failure (HF) patients even though it has been shown to relieve symptoms (p. 7) | Record daily weight (p. 4)  Limit sodium intake to <2000mg per day (p. 4)  The recommended total amount of fluid intake per day is 1.5-2L (p. 4)  Limit alcohol consumption to no more than 1 drink per day (p. 4)  Stop smoking (p. 4)  If stable symptoms and volume status, the goal is 30 minutes of continuous moderate exercise, and weight-bearing/resistance and flexibility activities at least twice a week (p. 4)  Annual influenza vaccine (p. 4) |
| 7. SIGN | 2016 | The NYHA classification (p. 8)  Weight gain (>2kg/week) (p. 14)  Paroxysmal nocturnal dyspnea/orthopnea (p. 14)  Nocturnal cough/wheezing (p. 14)  Ankle swelling, bloated feeling, peripheral edema (p. 14)  Confusion (especially in older people) (p. 14)  Syncope (p. 14)  Tachycardia (p. 14) | N/R | Opioids (p. 42)  No evidence was identified that oxygen at rest or when ambulatory is beneficial in patients with HF (p. 42)  Diuretic or BB (p. 70)  ACE-I (p. 25)  Intolerant of ACE-I, give ARB (p. 26)  Ongoing symptoms (NYHA class II-IV) with BB and ACE-I, add MRA (p. 31) | Strongly advise not to smoke and should be offered smoking cessation advice and support (p. 22)  Refrain from excessive alcohol consumption (p. 22)  Encourage patient to weigh themselves daily (after waking, before dressing, after voiding, before eating) (p. 70) |
| 8. Canadian Cardiovascular Society Heart Failure Management (Acute and Chronic Heart Failure) | 2012 | N/R | N/R | Loop diuretic (p. 176)  Nitrates can be useful to relieve dyspnea or angina but continuous use should be avoided because of risk tolerance development (p. 176)  Diuretics + ACE-I (if intolerant to ACE-I then ARB) + BB (p. 174)  MRAs (p. 174)  Digoxin (p. 174, p. 176) | N/R |
| 9. American College of Cardiology/American Heart Association | 2013 | NYHA (p. e155)  Rapid weight gain (p. e162).  Cachexia (p. e162) | N/R | Diuretics (p. e173)  Combination of hydralazine and isosorbide dinitrate who cannot be given an ACE-I or ARB (p. e179)  ACE-Is (p. 174)  ARBs who are ACE-I intolerant (p. 175)  BB (p. 176)  Omega-3 polyunsaturated fatty acid (PUFA) supplementation (p. 181) | Fluid restriction (1.5 – 2L/day). Sodium and fluid balance recommendations are best implemented in the context of weight and symptom monitoring programs (p. 190) |
| 10. American College of Cardiology/American Heart Association | 2016 | N/R | N/R | ACE-I (p. 9)  ARBs who are intolerant to ACE-I (pg. 9)  Replacement of ACE-I and ARB by an angiotensin receptor-neprilysin inhibitor (ARNI) (p. 10) | N/R |
| 11. National Heart Foundation of Australia (prevention, detection and management) | 2011 | N/R | N/R | Diuretics (p. 6)  Hydralazine-isosorbide dinitrate combination reserved for patients intolerant of ACE-Is and angiotensin II receptor antagonists (p. 31)  ACE-Is (p. 31)  BBs (pg. 31)  ARBs with spironolactone (p. 31)  Fish oil as a second-line agent (p. 31)  Nitrates (p. 44)  Digoxin (p. 29) | Fluid management (p. 22)  Excessive dietary sodium intake should be avoided (p. 22)  Alcohol intake should not exceed 1 – 2 drinks/day (p. 23)  Patients should not smoke or chew tobacco (p. 23)  Exercise/conditioning program (p. 32) |
| 12. European Society of Cardiology – Acute and Chronic Heart Failure | 2016 | Breathlessness, orthopnea, paroxysmal nocturnal dyspnea, reduced exercise tolerance, fatigue, tiredness, increased time to recover after exercise, ankle swelling, nocturnal cough, wheezing, bloated feeling, loss of appetite, confusion (especially in the elderly), depression, palpitations, dizziness, syncope, weight gain, weight loss, peripheral edema, tachycardia, irregular pulse (p. 2140) | N/R | ACE-I, in addition to a BB (pg. 2192)  MRA (p. 2192)  Diuretics (p. 2192)  Sacubitril/valsartan as a replacement for ACE-I (p. 2192) | Regular aerobic exercise (p. 2193)  Stop smoking and taking recreational substances (p. 2188)  Monitor body weight and prevent malnutrition (p. 2188)  Eat healthily, avoid excessive salt intake (>6g/day) and maintain a healthy body weight (p. 2188)  Fluid restriction of 1.5-2L/day (p. 2188)  Immunization against influenza and pneumococcal disease (p. 2188) |

Appendix B

Excluded studies. All citations listed below were reviewed in their full-text version and excluded for the reason indicated. An alphabetical reference list follows the table.

| **Citation** | **Not Systematic Review/Guideline** | **No Symptoms** | **Paediatric** | **Protocol Only** | **Pulmonary Hypertension** |
| --- | --- | --- | --- | --- | --- |
| Armenian 2015 |  | X |  |  |  |
| Armenian 2016 |  | X |  |  |  |
| Barac 2015 | X |  |  |  |  |
| Barthel 2016 |  |  | X |  |  |
| Bovelli 2010 |  | X |  |  |  |
| Chen 2016 |  | X |  |  |  |
| Conway 2015 |  | X |  |  |  |
| Demirci 2010 | X |  |  |  |  |
| Galiè 2015 |  |  |  |  | X |
| Harrison 2016 | X |  |  |  |  |
| Howlet 2013 |  | X |  |  |  |
| Kenyon 2014 | X |  |  |  |  |
| Lainscak 2011 | X |  |  |  |  |
| Lenneman 2016 | X |  |  |  |  |
| Mishra 2012 |  | X |  |  |  |
| Moe 2013 |  | X |  |  |  |
| Nathan 2016 | X |  |  |  |  |
| National Heart Foundation of Australia 2011 | X |  |  |  |  |
| Okada 2012 |  |  | X |  |  |
| Rushton 2015 |  |  |  | X |  |
| Schmitz 2012 | X |  |  |  |  |
| Steingart 2013 |  | X |  |  |  |
| Valachis 2015 | X |  |  |  |  |
| Virani 2016 |  | X |  |  |  |
| Walsh 2010 | X |  |  |  |  |
| Wong 2014 | X |  |  |  |  |
| Yun 2015 |  |  | X |  |  |
| Zamorano 2016 |  | X |  |  |  |

List of Excluded Studies

1. Armenian S., Hudson M., Mulder R., Chen M., Constine L., Dwyer M., Nathan P., Tissing W., Shankar S., Sieswerda E., Skinner R., Steinberger J., van Dalen E., van der Pal H., Wallace W., Levitt G., Kremer L. & International Late Effects of Childhood Cancer Guideline Harmonization Group. (2015). Recommendations for cardiomyopathy surveillance for survivors of childhood cancer: A report from the international late effects of childhood cancer guideline harmonization group. *The Lancet Oncology, 16*(3): e123 – e136. doi: 10.1016/S1470-2045(14)70409-7

2. Armenian S., Lacchetti C., Barac A., Carver J., Constine L., Denduluri N., Dent S., Douglas P., Durand J., Ewer M., Fabian C., Hudson M., Jessup M., Jones L., Ky B., Mayer E., Moslehi J., Oeffinger K., Ray K., Ruddy K. & Lenihan D. (2016). Prevention and monitoring of cardiac dysfunction in survivors of adults cancers: American society of clinical oncology clinical practice guideline. *Journal of Clinical Oncology, 35*(8): 893 – 911. doi: 10.1200/JCO.2016.70.5400

3. Barac A., Murtagh G., Carver J., Chen M., Freeman A., Herrmann J., Iliescu C., Ky B., Mayer E., Okwuosa T., Plana J., Ryan T., Rzeszut A. & Douglas P. (2015). Council clinical perspective: Cardiovascular health of patients with cancer and cancer survivors: A roadmap to the next level. *Journal of the American College of Cardiology, 65*(25): 2739 – 2746. doi: 10.1016/j.jacc.2015.04.059

4. Barthel E., Spencer K., Banco D., Kiernan E. & Parsons S. (2016). Is the adolescent and young adult cancer survivor at risk for late effects? It depends on where you look. *Journal of Adolescent and Young Adult Oncology, 5*(2): 159 – 173. doi: 10.1089/jayao.2015.0049

5. Bovelli D., Plataniotis G. & Roila F. (2010). Cardiotoxicity of chemotherapeutic agents and radiotherapy-related heart disease: ESMO clinical practice guidelines. *Annals of Oncology, 21*(Supplementary 5): v277 – v282. doi: 10.1093/annonc/mdq200

6. Chen J., Wu P.T., Middlekauff H. & Nguyen K.L. (2016). Aerobic exercise in anthracycline-induced cardiotoxicity: A systematic review of current evidence and future directions. *American Journal of Physiology. Heart and Circulatory Physiology.* doi: 10.1152/ajpheart.00646.2016

7. Conway A., McCarthy A., Lawrence P. & Clark R. (2015). The prevention, detection and management of cancer treatment-induced cardiotoxicity: A meta-review. *BioMed Central Cancer, 15*(366): 1 – 16. doi: 10.1186/s12885-015-1407-6

8. Demirci U., Benekli M., Buyukberber S. & Coskun U. (2010). Late side effects of cancer therapy. *International Journal of Hematology and Oncology, 4*(20): 250 – 261.

9. Galiè N., Humbert M., Vachiery J.L., Gibbs S., Lang I., Torbicki A., Simonneau G., Peacock A., Noordegraaf A., Beghetti M. & Ghofrani A. (2015). 2015 ESC/ERS guidelines for the diagnosis and treatment of pulmonary hypertension. *European Heart Journal, 37*(1): 67 – 119. doi: 10.1093/eurheartj/ehv317

10. Harrison J., Pressler S. & Friese C. (2016). Cardiotoxic heart failure in breast cancer survivors: A concept analysis. *Journal of Advanced Nursing, 72*(7): 1518 – 1528. doi: 10.1111/jan.12988

11. Howlett J. & MacFadyen J. (2013). Treatment of diabetes in people with heart failure. *Canadian Journal of Diabetes, 37*(2013): S126 – S128. doi: 10.1016/j.jcjd.2013.01.036

12. Kenyon M., Mayer D. & Owens A. (2014). Late and long-term effects of breast cancer treatment and surveillance management for the general practitioner. *Journal of Obstetric, Gynecologic & Neonatal Nursing, 43*(3): 382 – 398. doi: 10.1111/1552-6909.12300

13. Lainscak M., Blue L., Clark A., Dahlström U., Dickstein K., Ekman I., McDonagh T., McMurray J., Ryder M., Stewart S., Strömberg A. & Jaarsma T. (2011). Self-care management of heart failure: Practical recommendations from the patient care committee of the heart failure association of the European society of cardiology. *European Journal of Heart Failure, 13*(2): 115 – 126. doi: 10.1093/eurjhf/hfq219

14. Lenneman C. & Sawyer D. (2016). Cardio-oncology: An update on cardiotoxicity of cancer-related treatment. *Circulation Research, 118*(6): 1008 – 1020. doi: 10.1161/CIRCRESAHA.115.303633

15. Mishra S., Scherer R., Geigle P., Berlanstein D., Topaloglu O., Gotay C. & Snyder C. (2012). Exercise interventions on health-related quality of life for cancer survivors (review).*Cochrane Database of Systematic Reviews, 8*: CD007566. doi: 10.1002/14651858.CD007566.pub2

16. Moe G., Ezekowitz J., O’Meara E., Howlett J., Fremes S., Al-Hesayen A., Heckman G., Ducharme A., Estrella-Holder E., Grzeslo A., Harkness K., Lepage S., McDonald M., McKelvie R., Nilgam A., Rajda M., Rao V., Swiggum E., Virani S., Van Le V. & Zieroth S. (2013). The 2013 Canadian cardiovascular society heart failure management guidelines update: Focus on rehabilitation and exercise and surgical coronary revascularization. *Canadian Journal of Cardiology, 30*(2014): 249 – 263. doi: 10.1016/j.cjca.2013.10.010

17. Nathan P., Amir E., Abdel-Qadir H. (2016). Cardiac outcomes in survivors of pediatric and adult cancer. *Canadian Journal of Cardiology, 32*(2016): 871 – 880. doi: 10.1016/j.cjca.2016.02.065

18. National Heart Foundation of Australia. (2011). Diagnosis and management of chronic heart failure. Retrieved from https://www.heartfoundation.org.au/images/uploads/publications/CHF-QRG-updated-2014.pdf

19. Okada M., Meeske K., Menteer J. & Freyer D. (2012). Exercise recommendations for childhood cancer survivors exposed to cardiotoxic therapies: An institutional clinical practice initiative. *Journal of Pediatric Oncology Nursing, 29*(5): 246 – 252. doi: 10.1177/1043454212451525

20. Rushton M., Clark R., Brideson G. & Damarell R. (2015). The effectiveness of non-pharmacological interventions for the management of cancer treatment-induced cardiotoxicity: A systematic review protocol. *The Joanna Briggs Institute Database of Systematic Reviews & Implementation Reports, 13*(5): 53 – 73. doi: 10.11124/jbisrir-2015-2011

21. Schmitz K., Prosnitz R., Schwartz A. & Carver J. (2012). Prospective surveillance and management of cardiac toxicity and health in breast cancer survivors. *Cancer, 118*(8 suppl): 2270 – 2276. doi: 10.1002/cncr.27462

22. Steingart R., Yadav N., Manrique C., Carver J. & Liu J. (2013). Cancer survivorship: Cardiotoxic therapy in the adult cancer patient; cardiac outcomes with recommendations for patient management. *Seminars in Oncology, 40*(6): 690 – 708. doi: 10.1053/j.seminoncol.2013.09.010

23. Valachis A. & Nilsson C. (2015). Cardiac risk in the treatment of breast cancer: Assessment and management. *Breast Cancer (Dove Medical Press), 2015*(7): 21 – 35. doi: 10.2147/BCTT.S47227

24. Virani S., Dent S., Brezden-Masley C., Clarke B., Davis M., Jassal D., Johnson C., Lemieux J., Paterson I., Sebag I., Simmons C., Sulpher J., Thain K., Thavendiranathan P., Wentzell J. & Wurtele N. (2016). Canadian cardiovascular society guidelines for evaluation and management of cardiovascular complications of cancer therapy. *Canadian Journal of Cardiology, 32*(2016): 831 – 841. doi: 10.1016/j.cjca.2016.02.078

25. Walsh M. (2010). Impact of treatment-related cardiac toxicity on lymphoma survivors: An institutional approach for risk reduction and management. *Clinical Journal of Oncology Nursing, 14*(4): 505 – 507. doi: 10.1188/10.CJON.505-507

26. Wong D. & Hurvitz S. (2014). Cardiotoxicity of targeted agents in oncology: A medical oncology perspective. *Oncology (Williston Park), 28*(6): 490 – 492.

27. Yun S., Vincelette N. & Abraham I. (2015). Cardioprotective role of ß-blockers and angiotensin antagonists in early-onset anthracyclines-induced cardiotoxicity in adult patients: A systematic review and meta-analysis. *Postgraduate Medical Journal, 91*(1081): 627 – 633. doi: 10.1136/postgradmedj-2015-133535

28. Zamorano J., Lancellotti P., Muñoz D., Aboyans V., Asteggiano R., Galderisi M., Habib G., Lenihan D., Lip G., Lyon A., Fernandez T., Mohty D., Piepoli M., Tamargo J., Torbicki A. & Suter T. (2016). 2016 ESC position paper on cancer treatments and cardiovascular toxicity developed under the auspices of the ESC committee for practice guidelines. *European Heart Journal, 2016*(37): 2768 – 2801. doi: 10.1093/eurheartj/ehw211
